# Real-Time Hand Gesture Recognition Using Finger Segmentation

**DOI:** 10.1155/2014/267872

**Published:** 2014-06-25

**Authors:** Zhi-hua Chen, Jung-Tae Kim, Jianning Liang, Jing Zhang, Yu-Bo Yuan

**Affiliations:** ^1^Department of Computer Science and Engineering, East China University of Science and Technology, Shanghai 200237, China; ^2^State Key Laboratory for Novel Software Technology, Nanjing University, Nanjing 210093, China

## Abstract

Hand gesture recognition is very significant for human-computer interaction. In this work, we present a novel real-time method for hand gesture recognition. In our framework, the hand region is extracted from the background with the background subtraction method. Then, the palm and fingers are segmented so as to detect and recognize the fingers. Finally, a rule classifier is applied to predict the labels of hand gestures. The experiments on the data set of 1300 images show that our method performs well and is highly efficient. Moreover, our method shows better performance than a state-of-art method on another data set of hand gestures.

## 1. Introduction

As we know, the vision-based technology of hand gesture recognition is an important part of human-computer interaction (HCI). In the last decades, keyboard and mouse play a significant role in human-computer interaction. However, owing to the rapid development of hardware and software, new types of HCI methods have been required. In particular, technologies such as speech recognition and gesture recognition receive great attention in the field of HCI.

Gesture is a symbol of physical behavior or emotional expression. It includes body gesture and hand gesture. It falls into two categories: static gesture [1–4] and dynamic gesture [5–8]. For the former, the posture of the body or the gesture of the hand denotes a sign. For the latter, the movement of the body or the hand conveys some messages. Gesture can be used as a tool of communication between computer and human [9–11]. It is greatly different from the traditional hardware based methods and can accomplish human-computer interaction through gesture recognition. Gesture recognition determines the user intent through the recognition of the gesture or movement of the body or body parts. In the past decades, many researchers have strived to improve the hand gesture recognition technology. Hand gesture recognition has great value in many applications such as sign language recognition [12–15], augmented reality (virtual reality) [16–19], sign language interpreters for the disabled [20], and robot control [21, 22].

In [12, 13], the authors detect the hand region from input images and then track and analyze the moving path to recognize America sign language. In [23], Shimada et al. propose a TV control interface using hand gesture recognition. Keskin et al. [24] divide the hand into 21 different regions and train a SVM classifier to model the joint distribution of these regions for various hand gestures so as to classify the gestures. Zeng et al. [20] improve the medical service through the hand gesture recognition. The HCI recognition system of the intelligent wheelchair includes five hand gestures and three compound states. Their system performs reliably in the environment of indoor and outdoor and in the condition of lighting change.

The work flow of hand gesture recognition [25–27] is described as follows. First, the hand region is detected from the original images from the input devices. Then, some kinds of features are extracted to describe hand gestures. Last, the recognition of hand gestures is accomplished by measuring the similarity of the feature data. The input devices providing the original image information includes normal camera, stereo camera, and ToF (time of flight) camera. The stereo camera and ToF camera additionally provide the depth information so it is easy to segment the hand region from the background in terms of the depth map. For the normal camera, the skin color sensitive to the lighting condition and feature points are combined to robustly detect and segment the hand region. When the region of interest (ROI, the hand region in the case) is detected, features are needed to be extracted from the ROI region. Color, brightness, and gradient values are widely used features. Li and Kitani [28] describe various features for hand region detecting including the Gabor filter response, HOG, SIFT, BRIEF, and ORB. For the recognition of hand gestures, various classifiers, for example, SVM (support vector machine), HMM (hidden Markov model), CRF (conditional random field), and adapted boosting classifier are trained to discriminate hand gestures. Although the recognition performance of these sophisticated classifiers is good, the time cost is very high.

In this paper, we present an efficient and effective method for hand gesture recognition. The hand region is detected through the background subtraction method. Then, the palm and fingers are split so as to recognize the fingers. After the fingers are recognized, the hand gesture can be classified through a simple rule classifier.

The novelty of the proposed method is listed as follows.

- The first novelty of the proposed method is that the hand gesture recognition is based on the result of finger recognition. Therefore, the recognition is accomplished by a simple and efficient rule classifier instead of the sophisticated but complicated classifiers such as SVM and CRF.
- Some previous works need the users to wear data glove [29] to acquire hand gesture data. However, the special sensors of data glove are expensive and hinder its wide application in real life. In the work [25], the authors use TOF camera, that is, Kinect sensor, to capture the depth of the environment and a special tape worn across the wrist to detect hand region. Our approach only uses a normal camera to capture the vision information of the hand gesture meanwhile does not need the help of the special tape to detect hand regions.
- The third advantage of the proposed method is that it is highly efficient and fit for real-time applications.

The rest of the paper is organized as follows. In Section 2, the proposed method for hand gesture recognition is described in detail. In Section 3, the performance of our approach is evaluated on a data set of hand gestures. Then, our method is compared with a state-of-art method (FEMD) [25] on another data set of hand gestures.  Section 4 presents the conclusion and future works.

## 2. The Proposed Method for Hand Gesture Recognition

### 2.1. The Overview of the Method

**Figure 1:**
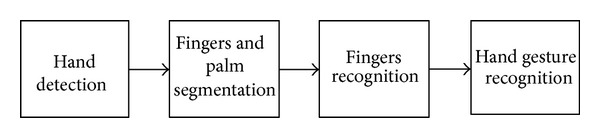
The overview of the proposed method for hand gesture recognition.

The overview of the hand gesture recognition is described in Figure 1. First, the hand is detected using the background subtraction method and the result of hand detection is transformed to a binary image. Then, the fingers and palm are segmented so as to facilitate the finger recognition. Moreover, the fingers are detected and recognized. Last, hand gestures are recognized using a simple rule classifier.

### 2.2. Hand Detection

**Figure 2:**
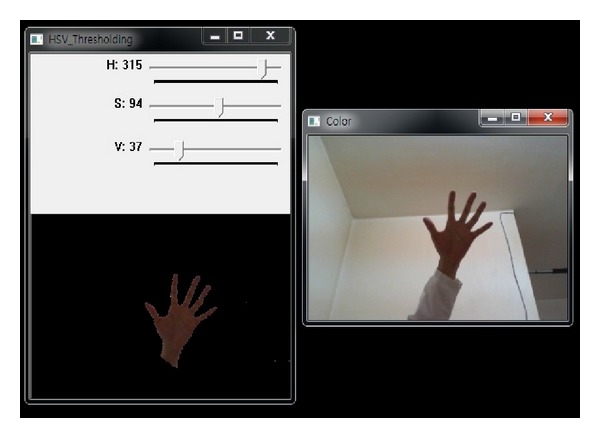
The procedure of hand detection.

The original images used for hand gesture recognition in the work are demonstrated in Figure 2. These images are captured with a normal camera. These hand images are taken under the same condition. The background of these images is identical. So, it is easy and effective to detect the hand region from the original image using the background subtraction method. However, in some cases, there are other moving objects included in the result of background subtraction. The skin color can be used to discriminate the hand region from the other moving objects. The color of the skin is measured with the HSV model. The HSV (hue, saturation, and value) value of the skin color is 315, 94, and 37, respectively. The image of the detected hand is resized to 200 × 200 to make the gesture recognition invariant to image scale.

### 2.3. Fingers and Palm Segmentation

**Figure 3:**
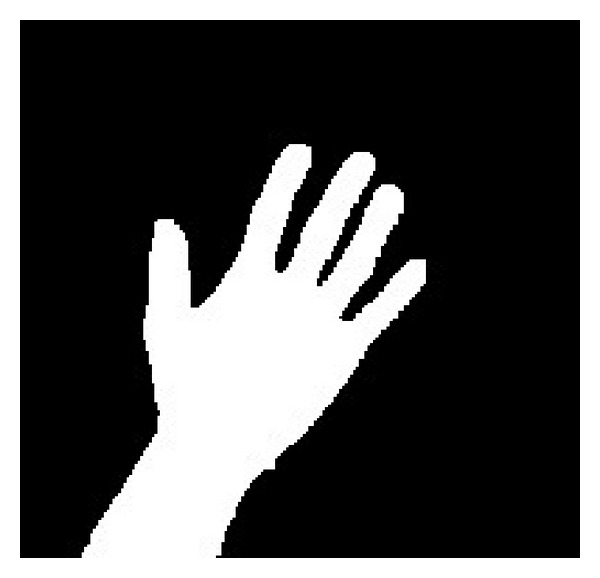
The detected hand region.

The output of the hand detection is a binary image in which the white pixels are the members of the hand region, while the black pixels belong to the background. An example of the hand detection result is shown in Figure 3. Then, the following procedure is implemented on the binary hand image to segment the fingers and palm.

**Figure 4:**
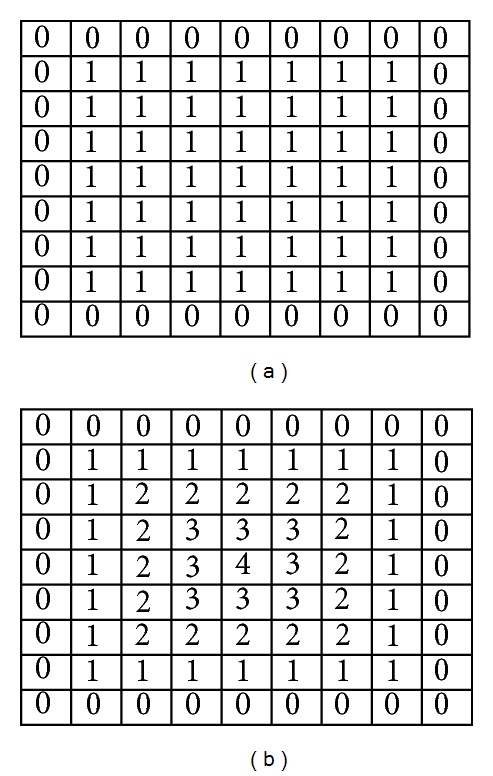
An example of distance transform: (a) is a binary image; (b) is the distance transform.

**Figure 5:**
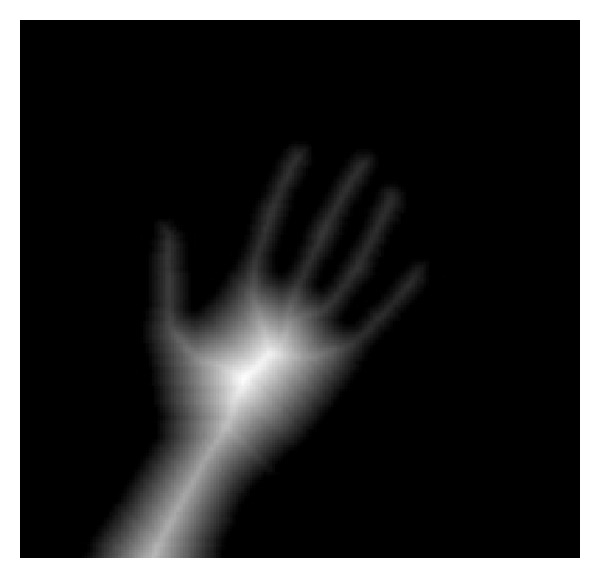
The distance transform of the hand image in Figure 3.

**Figure 6:**
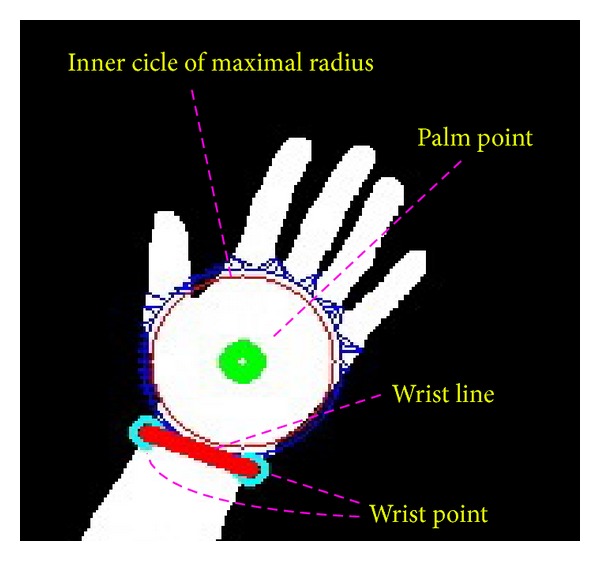
The palm point, wrist points, the wrist line, and the inner circle of the maximal radius.

*(i) Palm Point.* The palm point is defined as the center point of the palm. It is found by the method of distance transform. Distance transform also called distance map is a representation of an image. In the distance transform image, each pixel records the distance of it and the nearest boundary pixel. An example of distance transform is demonstrated in Figure 4. In Figure 4(a) is a binary image and in Figure 4(b) is the distance transform image. The block city distance is used to measure the distances between the pixels and the nearest boundary pixels. As is shown in the figure, the center point of the binary image is with the largest distance 4. Thus, in the distance transform image (refer to Figure 5) of the binary hand image, the pixel with largest distance is chosen as the palm point. The found palm point is marked with the point of the green color in Figure 6.

*(ii) Inner Circle of the Maximal Radius.* When the palm point is found, it can draw a circle with the palm point as the center point inside the palm. The circle is called the inner circle because it is included inside the palm. The radius of the circle gradually increases until it reaches the edge of the palm. That is the radius of the circle stops to increase when the black pixels are included in the circle. The circle is the inner circle of the maximal radius which is drawn as the circle with the red color in Figure 6.

*(iii) Wrist Points and Palm Mask.* When the radius of the maximal inner circle is acquired, a larger circle the radius of which is 1.2 times of that of the maximal inner circle is produced. The circle is drawn as the blue color circle in Figure 6. Then, some points (*X*, *Y*) are sampled uniformly along the circle. That is,

(1) X=Rcos⁡(θ∗π180)+X0,  Y=Rsin(θ∗π180)+Y0,θ=0:t:360,

where (*X*_0_, *Y*_0_) is the position of the palm point, *R* is the radius of the circle, and *t* is the sampling step.

**Figure 7:**
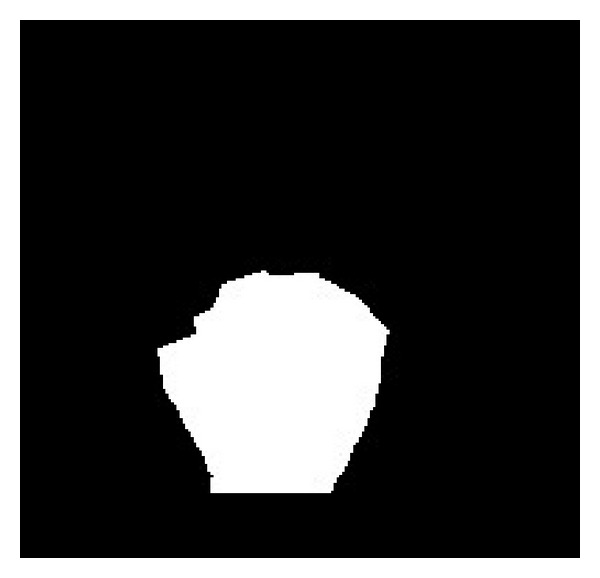
The palm mask.

For each sampled point on the circle, its nearest boundary point is found and lined to it. The boundary point is judged in a simple way. If the 8 neighbors of a pixel consist of white and black pixels, it is labeled as a boundary point. All of the nearest boundary points found are linked to yield the palm mask that can be used to segment fingers and the palm. The method for searching the palm mask is described in Algorithm 1. The palm mask of the hand image of Figure 3 is demonstrated in Figure 7. A larger circle instead of the maximal inner circle is used so as to yield a more accurate palm mask for the following segmentation.

Two wrist points are the two ending points of the wrist line across the bottom of the hand. The wrist points are important points for hand gesture recognition. They can be searched in the following manner: if the distance between two successive mask points *P*_*i*_, *P*_*i*+1_ are large, these two mask points are judged as the wrist points. That is,

(2) $$\begin{array}{c} \arg\underset{P_{i},P_{i+1}}{\max}\mathrm{dist}\left(P_{i},P_{i+1}\right),\;P_{i},P_{i+1}\in S, \end{array}$$

where *S* is the set of palm mask points and dist⁡(∗, ∗) is the distance between two points. Please refer to Figure 6 for the wrist points and wrist line.

**Figure 8:**
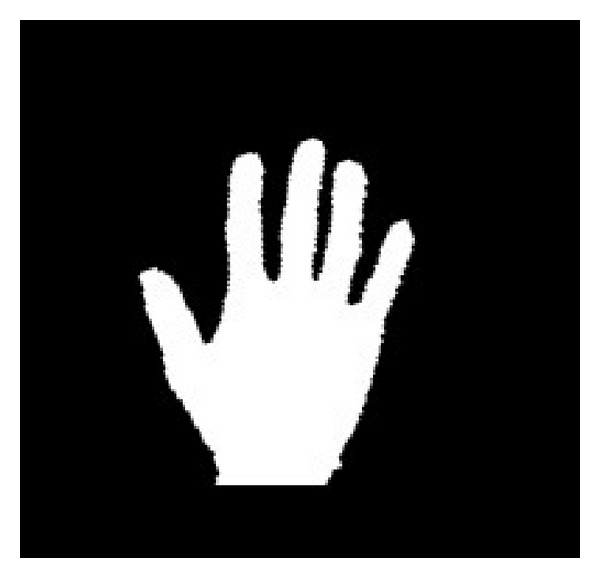
The rotated and cut hand image.

*(iv) Hand Rotation.* When the palm point and wrist point are obtained, it can yield an arrow pointing from the palm point to the middle point of the wrist line at the bottom of the hand. Then, the arrow is adjusted to the direction of the north. The hand image rotates synchronously so as to make the hand gesture invariant to the rotation. Meanwhile, the parts below the wrist line in the rotated image are cut to produce an accurate hand image that does not enclose the part of the arm.  Figure 8 is the rotated and cut hand image.

**Figure 9:**
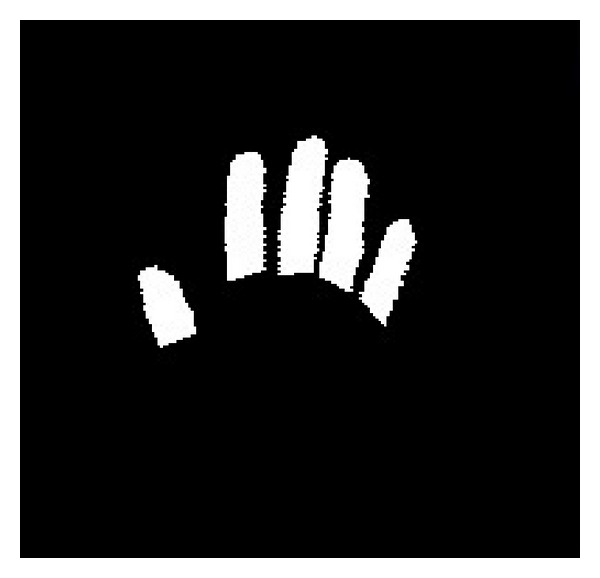
The segmented fingers.

*(v) Fingers and Palm Segmentation.* With the help of the palm mask, fingers and the palm can be segmented easily. The part of the hand that is covered by the palm mask is the palm, while the other parts of the hand are fingers. A segmentation result of fingers and the palm is shown in Figure 9.

### 2.4. Fingers Recognition

**Figure 10:**
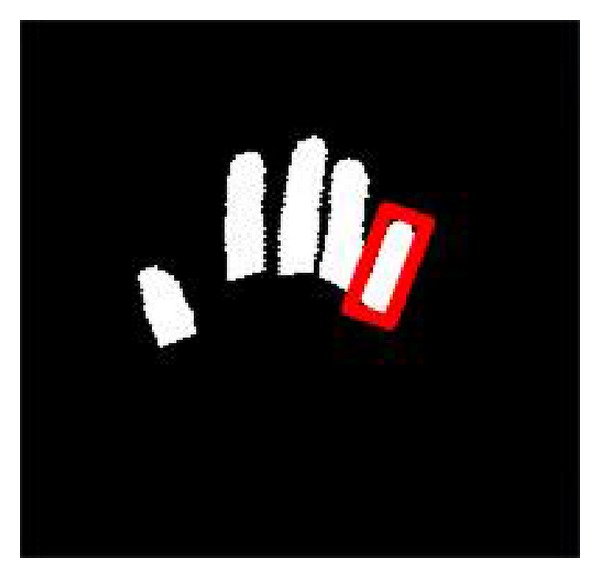
The minimal bounding box.

In the segmentation image of fingers, the labeling algorithm is applied to mark the regions of the fingers. In the result of the labeling method, the detected regions in which the number of pixels is too small is regarded as noisy regions and discarded. Only the regions of enough sizes are regarded as fingers and remain. For each remained region, that is, a finger, the minimal bounding box is found to enclose the finger. A minimal bounding box is denoted as a red rectangle in Figure 10. Then, the center of the minimal bounding box is used to represent the center point of the finger.

*(i) Thumb Detection and Recognition.* The centers of the fingers are lined to the palm point. Then, the degrees between these lines and the wrist line are computed. If there is a degree smaller than 50°, it means that the thumb appears in the hand image. The corresponding center is the center point of the thumb. The detected thumb is marked with the number 1. If all the degrees are larger than 50°, the thumb does not exist in the image.

**Figure 11:**
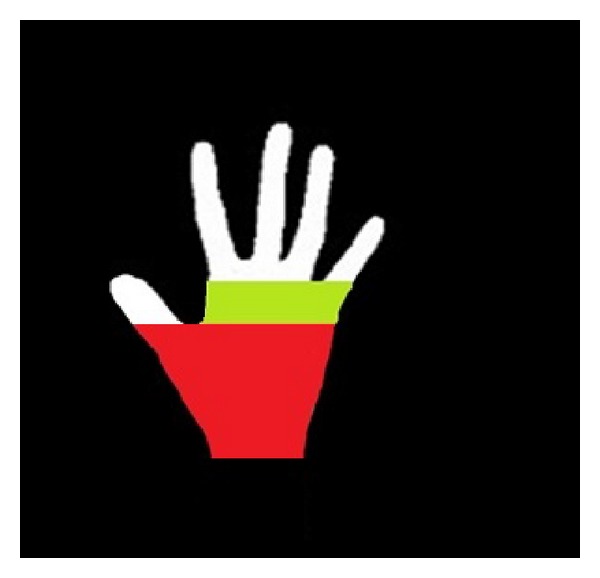
The palm line.

*(ii) Detection and Recognition of Other Fingers.* In order to detect and recognize the other fingers, the palm line is first searched. The palm line parallels to the wrist line. The palm line is searched in the way: start from the row of the wrist line. For each row, a line paralleling to the wrist line crosses the hand. If there is only one connected set of white pixels in the intersection of the line and the hand, the line shifts upward. Once there are more than one connected sets of white pixels in the intersection of the line and the hand, the line is regarded as a candidate of the palm line. In the case of the thumb not detected, the line crossing the hand with more than one connected sets of white pixels in their intersection is chosen as the palm line. In the case of the thumb existing, the line continues to move upward with the edge points of the palm instead of the thumb as the starting point of the line. Now, since the thumb is taken away, there is only one connected set of pixels in the intersection of the line and the hand. Once the connected set of white pixels turns to 2 again, the palm line is found. The search of the palm line is shown in Figure 11.

**Figure 12:**
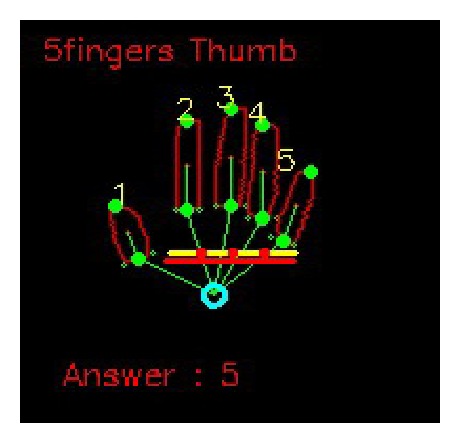
The recognition of the fingers.

After the palm line is obtained, it is divided into 4 parts. According to the horizontal coordinate of the center point of a finger, it falls into certain parts. If the finger falls into the first part, it is the forefinger. If the finger belongs to the second part, it is the middle finger. The third part corresponds to the ring finger. The fourth part is the little finger. The result of finger recognition of Figure 3 is demonstrated in Figure 12. In the figure, the yellow line is the palm line and the red line parallels to the wrist line.

In some case, two or more fingers stay closely and there is no interval among the fingers. An example of the case is referred to Figure 18. In order to discriminate the case from that of a single finger, the width of the minimal bounding box is used as a discrimination index. If the width of the minimal bounding box is equal to a usual value, the detected region is a single finger. If the width of the minimal bounding box is several times of the usual value, the detected region corresponds to several fingers that stay together closely. For the robustness of finger recognition, the distances and angles between fingers are also taken into account to discriminate different gestures.

### 2.5. Recognition of Hand Gestures

**Figure 13:**
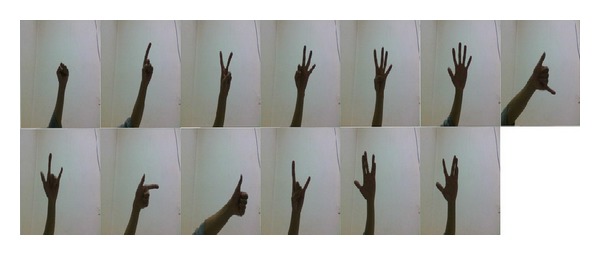
The image set of hand gestures used in the experiments. From left to right and then from top to bottom; these gestures are labeled as 0, 1, 2, 3, 4, 5, 6, 7, 8, 9, S1, S2, and S3.

When the fingers are detected and recognized, the hand gesture can be recognized using a simple rule classifier. In the rule classifier, the hand gesture is predicted according to the number and content of fingers detected. The content of the fingers means what fingers are detected. The rule classifier is very effective and efficient. For example, if three fingers, that is, the middle finger, the ring finger, and the little finger, are detected, the hand gesture is classified as the label 3 (refer to Figure 13 for the labels of the hand gestures).

## 3. Experimental Results

### 3.1. Data Sets

In the experiments, two data sets of hand gestures are used to evaluate the performance of the proposed method. The data set 1 is an image collection of thirteen gestures. For each gesture, 100 images are captured. So, there are total 1300 images for hand gesture recognition. All the gesture images belong to 3 females and 4 males. The size of one gesture image is 640 × 480. The thirteen gestures are shown in Figure 13. From left to right and then from top to bottom, these gestures are labeled as 0, 1, 2, 3, 4, 5, 6, 7, 8, 9, S1, S2, and S3.

Another data set [25] is collected from 10 subjects, and it contains 10 gestures for number 0 to 9. So, there are total 10 × 10 × 10 cases. The data set captured in cluttered backgrounds is a great challenge for hand gesture recognition. Besides, for each gesture, the subject poses with variations in hand orientation, scale, articulation, and so forth. We compare our method with FEMD [25] on the data set.

### 3.2. Performance Evaluation on Data Set 1

**Figure 14:**
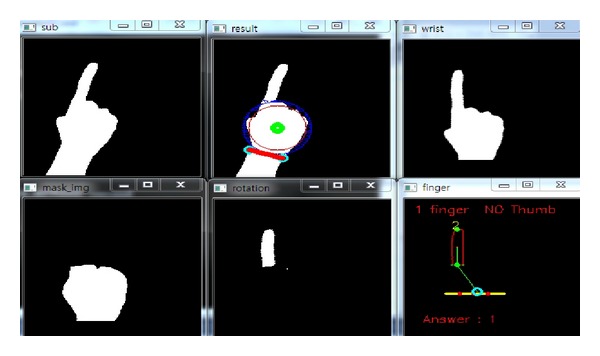
The recognition of the hand gesture 1.

**Figure 15:**
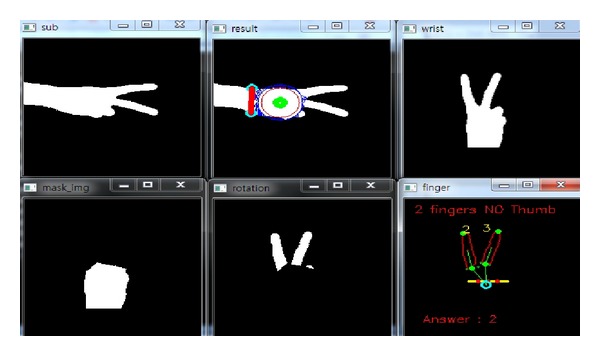
The recognition of the hand gesture 2.

**Figure 16:**
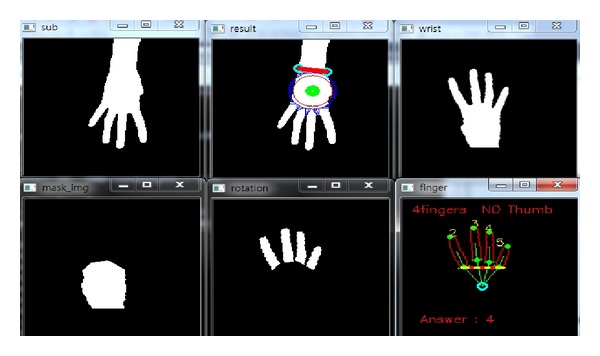
The recognition of the hand gesture 4.

**Figure 17:**
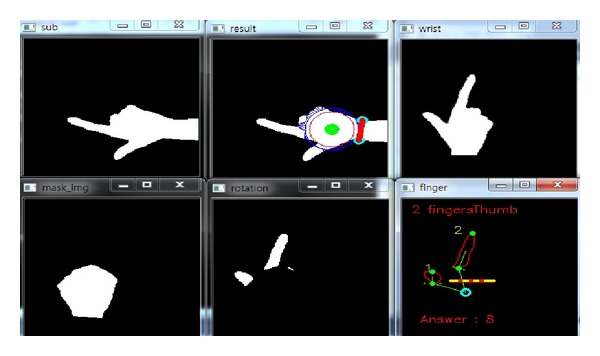
The recognition of the hand gesture 8.

**Figure 18:**
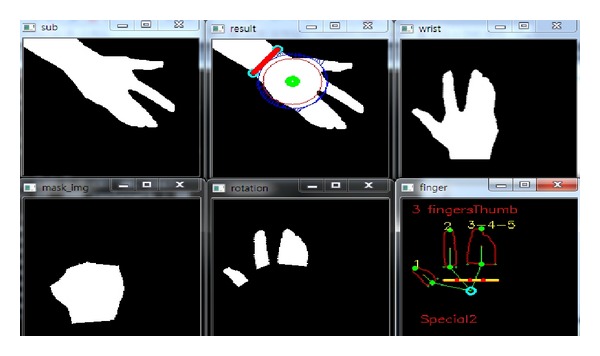
The recognition of the hand gesture S2.

*(i) Classification Accuracy.* In order to measure the performance of the proposed hand gesture recognition method, the classification accuracy is evaluated in the experiments. In the training stage, the rules discriminating the thirteen gestures are produced. Then, the rule classifier uses the rules to predict the identification of the testing image. In Figures 14, 15, 16, 17, and 18, the recognition of five gestures are demonstrated. In each figure, there are six subfigures which are the images showing the binary hand image, the palm point and wrist line, the calibrated hand image, the palm mask, the detected fingers, and finger and gesture recognition, respectively. In the subfigure of finger and gesture recognition, the label of the gesture is predicted. The predicted label is shown behind the word “Answer.”

The classification result of the total 1300 images is summarized with a confusion matrix in the Table 1. In the confusion matrix, the first column and the last row are the labels of the gestures. The other entries of the matrix record the numbers of the gesture images predicted as the corresponding labels. For example, for the first row, the numbers 99 and 1 are in the columns corresponding to the labels 1 and 3, respectively. It means that there are 99 and 1 images predicted as the labels 1 and 3 in the 100 testing images of the gesture 1. So, for the testing images of the gesture 1, the classification accuracy is 99%. As is shown in the confusion matrix, the proposed method performs well and obtains very high classification accuracies. The total classification accuracy of 1300 testing image is 96.69%. In the confusion matrix, the gestures of S2 and S3 are misclassified as 5. The reason is described as follows: for some gestures of S2 and S3, the fingers do not stay closely. That is, there is a hole between two fingers. So, in these cases, the gestures of S2 and S3 are misclassified as 5.

*(ii) Time Cost.* The time cost for recognizing the gestures is reported in Table 2. In the table, the unit of the time cost is second. A value in the second row is the averaging runtime of 100 images of one gesture. For the total 1300 images, the averaging time cost to recognize hand gestures is 0.024 seconds. The experiments are run on the laptop computer of Intel i7-2630 2.00 GHz CPU and 4 GB RAM. It is obvious that the proposed method is very highly efficient and can meet the requirement of the real-time applications.

### 3.3. Performance Comparison on Data Set 2

The comparison of the proposed method and a state-of-art method FEMD is performed on data set 2. The classification results are also summarized with the confusion matrixes. The description of the confusion matrixes is similar to that in Table 1. The confusion matrix of our method is shown in Table 3. The confusion matrix of FEMD is demonstrated in Table 4. The averaging accuracy of the proposed method is 96.6%. The averaging accuracy of FEMD is 93.2%. The comparison results on data set 2 show that our method outperforms FEMD. The averaging time of our method spent on recognizing a hand gesture is 0.0202 seconds.

## 4. Conclusion and Future Works

A new method for hand gesture recognition is introduced in this paper. The hand region is detected from the background by the background subtraction method. Then, the palm and fingers are segmented. On the basis of the segmentation, the fingers in the hand image are discovered and recognized. The recognition of hand gestures is accomplished by a simple rule classifier. The performance of our method is evaluated on a data set of 1300 hand images. The experimental results show that our approach performs well and is fit for the real-time applications. Moreover, the proposed method outperforms the state-of-art FEMD on an image collection of hand gestures.

The performance of the proposed method highly depends on the result of hand detection. If there are moving objects with the color similar to that of the skin, the objects exist in the result of the hand detection and then degrade the performance of the hand gesture recognition. However, the machine learning algorithms can discriminate the hand from the background. ToF cameras provide the depth information that can improve the performance of hand detection. So, in future works, machine learning methods and ToF cameras may be used to address the complex background problem and improve the robustness of hand detection.

## Figures and Tables

**Algorithm 1:**
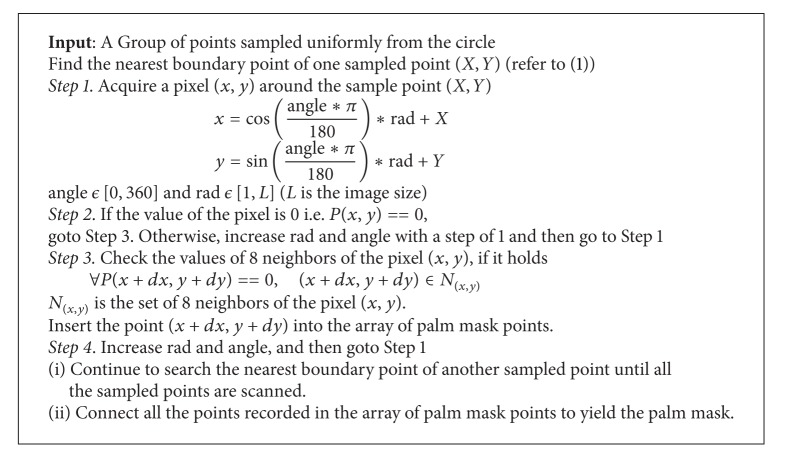
The method of producing the palm mask.

**Table 1:** The confusion matrix of hand gesture recognition on data set 1.

1	99		1										
2		94	6										
3		2	95	3									
4			4	95	1								
5				3	93	4							
6						100							
7	4						96						
8	2		5					92	1				
9									100				
0	1									99			
S1			1								99		
S2					2							98	
S3					3								97
	1	2	3	4	5	6	7	8	9	0	S1	S2	S3

**Table 2:** The runtime of hand gesture recognition.

1	2	3	4	5	6	7	8	9	0	S1	S2	S3
0.024	0.021	0.022	0.024	0.027	0.023	0.026	0.022	0.025	0.022	0.022	0.026	0.021

**Table 3:** The confusion matrix of our method on data set 2.

0	99	1								
1		100								
2		7	91					1		1
3				100						
4					99		1			
5					3	97				
6							99	1		
7			2	1			9	88		
8		7							93	
9										100
	0	1	2	3	4	5	6	7	8	9

**Table 4:** The confusion matrix of FEMD on data set 2 (from [25]).

0	95	1						3	1	
1	3	86	4	2			1	4		
2		2	94	2			2			
3			4	87	6		3			
4				7	89	3	1			
5	1	2				95			2	
6			1			1	96	2		
7	6	2						92		
8	1					1			98	
9										100
	0	1	2	3	4	5	6	7	8	9
